# Association of preoperative clinical frailty and clinical outcomes in elderly patients with stable coronary artery disease after percutaneous coronary intervention

**DOI:** 10.1007/s00380-023-02276-3

**Published:** 2023-06-07

**Authors:** Hirokazu Shimono, Akihiro Tokushige, Daisuke Kanda, Ayaka Ohno, Masao Hayashi, Mana Fukuyado, Mitsumasa Akao, Mariko Kawasoe, Ryo Arikawa, Hideaki Otsuji, Hideto Chaen, Hideki Okui, Naoya Oketani, Mitsuru Ohishi

**Affiliations:** 1 Department of Cardiovascular Medicine, Kagoshima City Hospital, Kagoshima, Japan; 2 Department of Cardiovascular Medicine and Hypertension, Graduate School of Medical and Dental Sciences, Kagoshima University, Kagoshima, Japan; 3 Department of Prevention and Analysis of Cardiovascular Diseases, Graduate School of Medical and Dental Sciences, Kagoshima University, 8-35-1 Sakuragaoka, Kagoshima, 890-8520 Japan; 4 Department of Clinical Pharmacology and Therapeutics, University of the Ryukyus School of Medicine, Nishihara, Okinawa Japan

**Keywords:** Clinical frailty scale, Stable coronary artery disease, Percutaneous coronary intervention, Clinical outcome

## Abstract

There are few reports on the long-term clinical outcome after percutaneous coronary intervention (PCI) in patients with stable coronary artery disease (CAD) complicated with frailty. This novel study investigated the association between pre-PCI frailty and long-term clinical outcomes in elderly patients aged 65 years or older with stable CAD who underwent elective PCI. We assessed 239 consecutive patients aged 65 years or older with stable CAD who underwent successful elective PCI at Kagoshima City Hospital between January 1st, 2017 and December 31st, 2020. Frailty was retrospectively assessed using the Canadian Study and Aging Clinical Frailty Scale (CFS). Based on the pre-PCI CFS, patients were divided into two groups: the non-frail (CFS < 5) and the frail (CFS ≥ 5) group. We investigated the association between pre-PCI CFS and major adverse cardiovascular events (MACEs) defined as the composite of all-cause death, non-fatal myocardial infarction, non-fatal stroke, and heart failure requiring hospitalization. Additionally, we assessed the association between pre-PCI CFS and major bleeding events defined as Bleeding Academic Research Consortium (BARC) type 3 or 5 bleeding. The mean age was 74.8 ± 7.0 years, and 73.6% were men. According to the pre-PCI frailty assessment, 38 (15.9%) and 201 (84.1%) were classified as frail and non-frail groups, respectively. During a median follow-up of 962 (607–1284) days, 46 patients developed MACEs and 10 patients developed major bleeding events. Kaplan–Meier curves showed a significantly higher incidence of MACE in the frail group compared to those in the non-frail group (Log-rank *p* < 0.001). Even in multivariate analysis, pre-PCI frailty (CFS ≥ 5) was independently associated with MACE (HR 4.27, 95% CI 1.86–9.80, *p*-value: < 0.001). Additionally, the cumulative incidence of major bleeding events was significantly higher in the frail group than in the non-frail group (Log-rank *p* = 0.001). Pre-PCI frailty was an independent risk factor for MACE and bleeding events in elderly patients with stable CAD who underwent elective PCI.

**Supplementary Information:**

The online version contains supplementary material available at 10.1007/s00380-023-02276-3.

## Introduction

Cardiovascular disease (CVD) is the leading cause of death among adults worldwide [1, 2]. In 2018, more than three hundred thousand people in Japan died of CVD, accounting for nearly one-fourth of all-cause deaths [3]. Recently, among CVD, coronary artery disease (CAD) based on atherosclerosis assumes clinical importance. Optimal medical therapy such as calcium channel blockers, nitrates, β-blocker, nicorandil, lipid-lowering therapy, and antiplatelet agents are part of well-established treatment protocols for stable CAD. Whereas revascularization therapy including percutaneous coronary intervention (PCI) and coronary artery bypass grafting (CABG) is believed to improve the clinical outcomes of stable CAD patients. However, to date, no randomized controlled trial has demonstrated a prognostic benefit of PCI against optimal medical therapy in stable CAD patients [4, 5]. A previous study has compared the short-term prognostic efficacy and relief of symptoms of revascularization therapy (PCI or CABG) with optimal medical therapy in elderly stable CAD patients [6]. However, whether the addition of revascularization therapy to optimal medical therapy in patients with stable CAD improves clinical outcomes were not fully elucidated. Reports indicate that elderly CAD patients have greater absolute risk reductions associated with revascularization therapy than younger CAD patients [7]. However, a Japanese prospective observational study revealed that procedural success rates and in-hospital adverse events did not differ between elderly (≥ 80 years old) and non-elderly (< 80 years old) CAD patients for elective PCI. Furthermore, the presence of comorbidities (such as left ventricular dysfunction and previous intracranial bleeding) had a stronger impact on worse clinical outcomes than age [8]. Thus, when considering the treatment of patients with stable CAD, clinical outcomes may be improved by selecting and treating patients who benefit greatly from revascularization, such as elderly patients with fewer comorbidities. Additionally, patient evaluation before PCI assumes more importance.

Frailty is indicative of vulnerability which increases the risk of adverse health or death when exposed to stressors [9]. The prevalence of frailty in cardiovascular disease ranges from 10 to 60%, and a twofold or more relative increase in frailty enhances mortality and morbidity risk than in non-frail patients [10]. The assessment of frailty in patients with cardiovascular disease is important and may be measured using many tools for frail assessment [11]; however, there is confusion regarding the choice of tool. Canadian Study and Aging Clinical Frailty Scale (CFS) is a simple tool to assess frailty in daily clinical practice [12]. Although previous studies have established that frailty defined by using CFS was associated with a poor prognosis of chronic heart failure [13] and acute coronary syndromes [14], only a few studies have investigated the clinical outcome and frailty with that of stable CAD patients who underwent elective PCI. Moreover, it has been reported that as the CFS increased, the patients at high bleeding risk (HBR) assessed by the Academic Research Consortium for High Bleeding Risk (ARC-HBR) criteria [15] and the Japanese version of HBR (J-HBR) criteria [16] were also increased, and increasing major bleeding events [17]. We hypothesized that stable CAD patients who are frail, as assessed by CFS, would have poorer clinical outcomes including major bleeding events than stable CAD patients without frailty. Although the Asia–Pacific Clinical Practice Guidelines for the Management of Frailty recommended that frailty should be routinely screened for adults aged 70 years and older [18], previous reports indicate a relatively high prevalence of frailty in those aged 65 years and older (4–16%) [19, 20]. Furthermore, the estimated prevalence of frail in Japanese elderly patients (65 years or older) was 8.7%, especially higher at 10.7% in Kyusyu and Okinawa [21]. Hence, we assessed frailty in those aged 65 years and older. This novel study investigates the association between frailty assessed by CFS and clinical outcomes including major bleeding events in elderly (≥ 65 years old) patients with stable CAD after elective PCI.

## Materials and methods

### Study design and settings

**Fig. 1:**
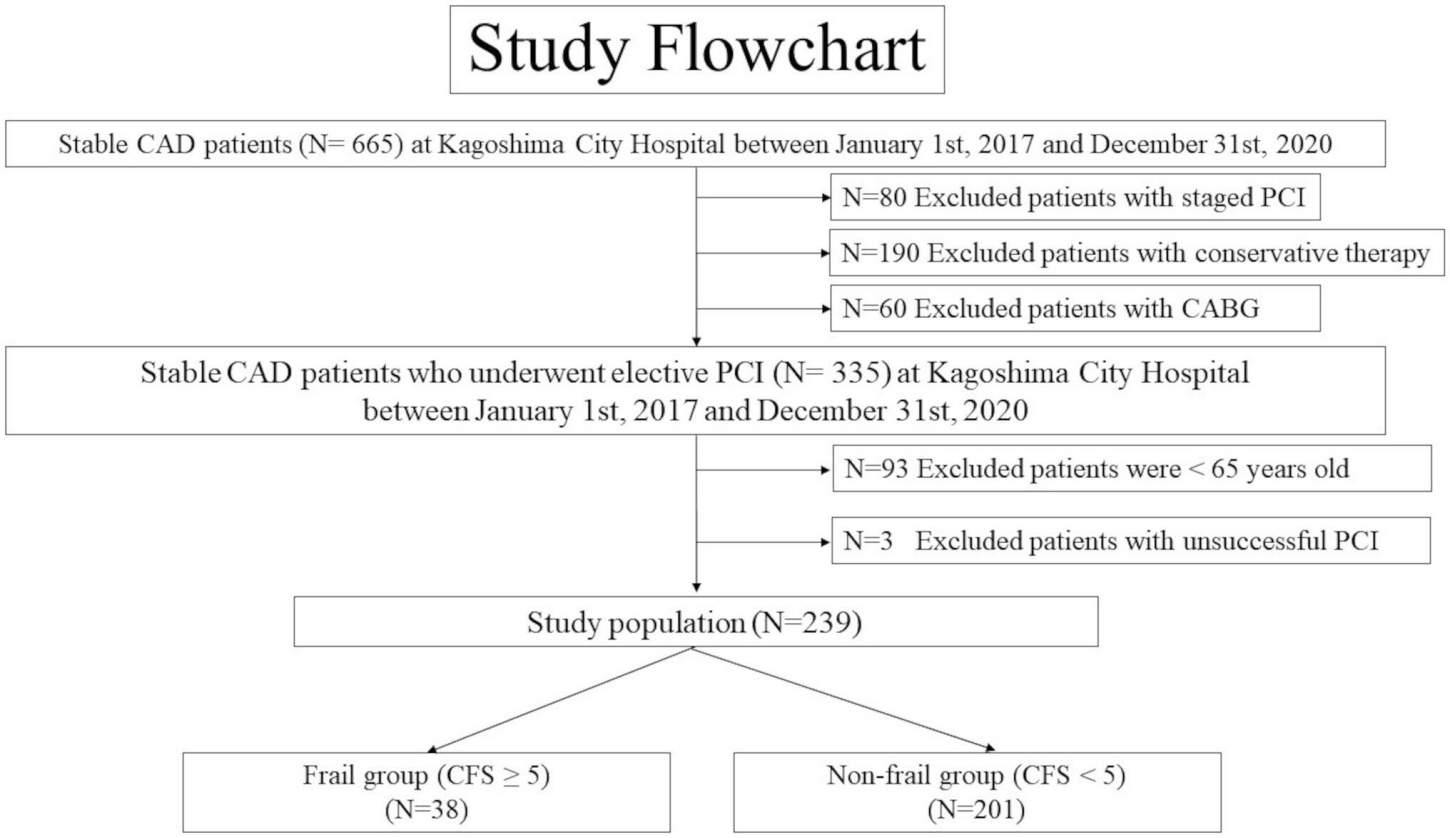
The study flowchart shows the patient selection. *CAD* coronary artery disease, *N* number, *PCI* percutaneous coronary intervention, *CABG* coronary artery bypass grafting, *CFS* clinical frailty scale

In this single-center, retrospective, observational study, after excluding 80 patients with staged PCI within three months after acute coronary syndrome, 190 patients with conservative therapy, and 60 patients with CABG for stable CAD, we reviewed consecutive 335 stable CAD patients who underwent elective PCI at Kagoshima City Hospital between January 1st, 2017 and December 31st, 2020, with the observation period until March 31, 2022. We excluded 93 patients with aged less than 65 years and three patients with unsuccessful PCI. Thus, consecutive 239 stable CAD patients who underwent successful elective PCI were included in the present study (Fig. 1). All patients underwent elective PCI for culprit lesions mainly using a new-generation drug-eluting stent (DES) with standard intracoronary imaging. In principle, indication for PCI of the stenotic or occluded lesion was defined as the following: 1. stenotic lesion ≥ 90% that causes stable effort angina; 2. stenotic lesion > 50% that is tested to evaluate functional ischemia (e.g., fractional flow reserve, instantaneous wave-free ratio, scintigraphy, exercise stress testing) and is regarded as the functional ischemic cause. Before elective PCI, all patients were administrated dual-antiplatelet therapy (DAPT). Patients who were hospitalized more than once during the study period were not double-counted and the data from the first hospitalization alone was employed in the analysis.

### Definitions

Stable CAD was defined as a chronic coronary syndrome (CCS) subtypes 1, 2, 3, 4 and 6 in the European Society of Cardiology guidelines published in 2019 [22]; however, patients with angina with coronary spasm or microangiopathy (CCS subtype 5) were not included. Hypertension was defined as systolic blood pressure ≥ 140 mmHg, diastolic blood pressure ≥ 90 mmHg, or usage of current antihypertensive medication. Diabetes mellitus (DM) was characterized by the use of anti-hyperglycemic medication or a previous diagnosis of diabetes mellitus, or glycated hemoglobin ≥ 6.5% (National Glycohemoglobin Standardization Program). Dyslipidemia was described as low-density lipoprotein cholesterol ≥ 140 mg/dL, high-density lipoprotein cholesterol < 40 mg/dL, triglycerides ≥ 150 mg/dL, or the use of current lipid-lowering medication. A current smoker was defined as a person with smoking habits at the time of admission. Chronic kidney disease (CKD) was specified as an estimated glomerular filtration rate (eGFR) < 60 mL/min/1.73 m^2^. Staged PCI was defined as scheduled PCI performed within 3 months of the initial PCI [23].

### Ethics approval

This study was conducted in accordance with the ethical standards laid down in the 1964 Declaration of Helsinki and its later amendments. It was approved by the institutional review board of Kagoshima City Hospital (registration number 2021-38). Written informed consent was obtained from all patients for the elective PCI, and informed consent for this study was obtained in the form of opt-out.

## Frailty assessment

Frailty was assessed using the Canadian Study of Health and Aging Clinical Frailty Scale score [12, 24] and was stratified from 1 (very fit) to 9 (terminally ill). Stable CAD patients were classified into two groups CFS < 5 (non-frail group) or CFS ≥ 5 (frail group). A CFS score of ≥ 5 indicates dependence on others for the key activities of daily living. The review article on the CFS revealed that in 68.9% of the studies about the CFS, a CFS score of five was the most widely used frailty cut-off point [25]. From the definition of CFS [12, 24], CFS4 is very mild frailty or vulnerable, and we considered that explicit frailty should be defined as CFS5 or higher. At the time of admission, nurses and physicians interviewed the patient and their relatives and recorded the pre-admission life history of the patient, including daily activities. The assessment of CFS data was retrospectively performed by trained investigators (physicians and clinical nursing staff) who were masked to the clinical presentation of the patient and outcome.

### Bleeding risk scores

To investigate the relationship between frailty and bleeding risk, we scored the ARC-HBR and J-HBR criteria, which was calculated by assigning 1 point to each ARC-HBR and J-HBR major criterion and 0.5 points to each ARC-HBR and J-HBR minor criterion. Patients were defined as HBR if they had an ARC-HBR score ≥ 1 or J-HBR score ≥ 1.

### Data collection and clinical outcome measures

Clinical and follow-up data were retrospectively obtained from the medical records at the time of the outpatient visit or by telephone interviews with patients or by obtaining data from the primary physician. The primary outcome measure was the cumulative incidence of a major adverse cardiovascular event (MACE) which was defined as the composite of all-cause death, non-fatal myocardial infarction (MI), non-fatal stroke, and heart failure requiring hospitalization. The secondary outcome measure was the cumulative incidence of major bleeding events defined as Bleeding Academic Research Consortium (BARC) type 3 or 5 bleeding and ischemic events defined as MI and ischemic stroke. We investigated the association of pre-PCI CFS with MACE, major bleeding, and ischemic events.

### Statistical analyses

Continuous variables were presented as means ± SD or median and interquartile range per data distribution. Categorical variables were presented as numbers and percentages. Non-normally distributed continuous variables were compared using the Mann–Whitney *U* test and normally distributed continuous variables were compared using the Student’s *t* test. Categorical variables were compared using the Chi-square test or Fisher’s exact test as appropriate. To investigate the relationship between frailty and bleeding risk, the correlation between the J-HBR score and CFS was evaluated using the Spearman correlation coefficient. Univariate and multivariate logistic regression analysis was used to assess factors associated with frailty (CFS ≥ 5). The cumulative incidence of MACE, major bleeding, and ischemic events was estimated using Kaplan–Meier analysis and compared between groups with the log-rank test. Subgroup analyses stratified by age (65–74 years and ≥ 75 years) were calculated in the same method. Univariate and multivariate analyses using the Cox proportional hazards regression model were employed in estimating the hazard ratios (HRs) and 95% confidence intervals (95%CI) of frail (CFS ≥ 5) on the primary outcomes. The clinically relevant factors adjusted in the multivariate analysis for MACE included age, sex, DM, left ventricular ejection fraction (LVEF) < 40%, serum albumin < 3.5 g/dL, and lesion location of left main coronary artery (LMCA) or multivessel disease. Reports indicate these factors to be predictors of worse clinical outcomes in patients with CAD [26–30]. Three different models were constructed, each incorporating adjustments for different variables. Model 1 was adjusted for age and sex, and Model 2 was adjusted for age, sex, DM, and serum albumin < 3.5 g/dL. Model 3 was adjusted for age, sex, DM, LVEF < 40%, LMCA or multivessel disease, and serum albumin < 3.5 g/dL. A *p*-value of less than 0.05 was considered statistically significant. All statistical analyses were performed using JMP Pro 15.0 software (SAS Institute Inc., Cary, NC, USA) and EZR (Saitama Medical Center, Jichi Medical University, Saitama, Japan; http://www.jichi.ac.jp/saitama-sct/SaitamaHP.files/statmedEN.html; Kanda, 2012), which is a graphical user interface for R (The R Foundation for Statistical Computing, Vienna, Austria, version 4.1.2). It is a modified version of R commander (version 2.7-1) designed to add statistical functions frequently used in biostatistics [31].

## Results

### Clinical characteristics and CFS

Table 1 indicates the baseline clinical characteristics of patients between the frail group (CFS ≥ 5) and the non-frail group (CFS < 5). According to the pre-PCI CFS assessment, 38 (15.9%) and 201 (84.1%) patients were classified as frail and non-frail. The average age was significantly higher in the frail group than in the non-frail group. However, there were no significant differences in sex, and coronary risk factor prevalence such as hypertension, DM, dyslipidemia, and current smoking between the two groups. More patients in the frail group had a history of heart failure, stroke, hemodialysis, and peripheral artery disease than in the non-frail group. Regarding laboratory findings, serum albumin, and hemoglobin levels were significantly lower, and eGFR was lower in the frail group compared with those in the non-frail group. In echocardiography findings, the proportion of reduced LVEF (LVEF < 40%) was significantly higher in the frail group than in the non-frail group. HBR patients defined by ARC-HBR and J-HBR scores were significantly higher proportions in the frail group than in the non-frail group. Furthermore, CFS and the J-HBR score showed a slightly positive correlation (*r* = 0.449, *p* < 0.001, Online Fig. 1). In terms of medication at discharge, statin was used commonly in the non-frail group, whereas anticoagulant usage was popular in the frail group. Baseline lesion and procedural characteristics between the frail and non-frail groups are shown in Table 2. In the indication of the PCI between the 2 groups, functional ischemia assessment was more common in the non-frail group than in the frail group. There were no significant differences in the proportions of finalized devices (DES, drug-coated balloon, and others), PCI access site, lesion location, and the number of diseased vessels between the two groups. On univariate and multivariate logistic regression analysis (Online Table 1), advanced age (≥ 75 years) (odd ratio [OR] 7.87, 95% CI 2.60–23.86: *p*-value < 0.001), history of stroke (OR 5.63, 95% CI 1.82–17.41: *p*-value = 0.003), LVEF < 40% (OR 5.49, 95% CI 1.51–19.86: *p*-value = 0.01), and serum albumin < 3.5 g/dL (OR 11.06, 95% CI 3.55–34.49: *p*-value < 0.001) were independently associated with frail (CFS ≥ 5).

**Table 1:** Baseline clinical characteristics

	Frail group (CFS ≥ 5)	Non-frail group (CFS < 5)	*p*-value
	(*N* = 38)	(*N* = 201)	
Age, years	79.5 ± 7.5	74.0 ± 6.6	< 0.001
Male sex	26 (68.4%)	150 (74.6%)	0.43
BMI (kg/m^2^)	22.0 (19.5–24.7)	23.2 (21.0–25.3)	0.049
BMI < 18.5 (kg/m^2^)	6 (15.8%)	18 (9.0%)	0.20
Coronary risk factors			
HTN	27 (71.1%)	165 (82.1%)	0.12
DM	20 (52.6%)	93 (46.3%)	0.47
Dyslipidemia	25 (65.8%)	149 (74.1%)	0.29
Current Smoker	8 (21.1%)	37 (18.4%)	0.70
Family history of CAD	2 (5.3%)	27 (13.4%)	0.16
History of heart failure	12 (31.6%)	28 (13.9%)	0.01
History of PCI	11 (29.0%)	68 (33.8%)	0.56
History of OMI	15 (39.5%)	67 (33.3%)	0.47
History of CABG	4 (10.5%)	15 (7.5%)	0.52
History of stroke	13 (34.2%)	28 (13.9%)	0.002
PAD	14 (36.8%)	31 (15.4%)	0.002
Hemodialysis	8 (21.1%)	12 (6.0%)	0.002
Atrial fiblliration	9 (23.7%)	23 (11.4%)	0.06
COPD	7 (18.4%)	21 (10.5%)	0.16
Laboratory data			
LDL-C (mg/dL)	74 (64–95)	85 (66–109)	0.08
HDL-C (mg/dL)	45 (37–50)	48 (39–58)	0.08
TG (mg/dL)	95 (66–116)	103 (76–140)	0.08
HbA1c (%)	6.1 (5.6–7.3)	6.1 (5.6–7.2)	0.90
eGFR(mL/min/1.73m^2^)	52 (31–72)	60 (49–71)	0.052
eGFR < 60 mL/min/1.73m^2^	24 (63.2%)	98 (48.8%)	0.10
Hemoglobin (g/dL)	11.7 (10.4–12.6)	12.9 (12.0–14.1)	< 0.001
Hemoglobin < 11 g/dL	13 (34.2%)	29 (14.4%)	0.006
Albumin (g/dL)	3.5 (3.0–3.8)	4.1 (3.8–4.3)	< 0.001
LVEF (%)	62 (39–70)	66 (55–72)	0.053
LVEF < 40%	10 (26.3%)	13 (6.5%)	< 0.001
HBR (ARC-HBR score ≥ 1)	37 (97.4%))	120 (59.7%)	< 0.001
ARC-HBR score	2.25 (2.0–3.0)	1.0 (0.5–1.5)	< 0.001
HBR (J-HBR score ≥ 1)	38 (100%)	146 (72.6%)	< 0.001
J-HBR score	3.5 (3.0–5.0)	1.5 (0.5–2.5)	< 0.001
Medication at discharge			
Aspirin	36 (94.7%)	198 (98.5%)	0.14
Thienopyridine			
Prasugrel	13 (34.2%)	99 (49.3%)	0.20
Clopidogrel	25 (65.8%)	101 (50.3%)	
Anticoagulant	14 (36.8%)	20 (10.0%)	< 0.001
Statin	30 (79.0%)	186 (92.5%)	0.009
Ezetimibe	7 (18.4%)	37 (18.4%)	0.99
Insulin	4 (10.5%)	26 (12.9%)	0.68
ACE-I/ARB	22 (57.9%)	121 (60.2%)	0.79
CCB	17 (44.7%)	120 (59.7%)	0.09
β-blocker	17 (44.7%)	83 (41.3%)	0.69
Proton pump inhibitor	33 (86.8%)	178 (88.6%)	0.76

Data are shown as mean ± standard deviation or median with interquartile range, and *n* (%)

*BMI* body mass index, *HTN* hypertension, *DM* diabetes mellitus, *CAD* coronary artery disease, *PCI* percutaneous coronary intervention, *OMI* old myocardial infarction, *CABG* coronary artery bypass grafting, *PAD* peripheral artery disease, *COPD* chronic obstructive pulmonary disease, *LDL-C* low-density lipoprotein-cholesterol, *HDL-C* high-density lipoprotein-cholesterol, *TG* triglyceride, *eGFR* estimated glomerular filtration rate, *LVEF* left ventricular ejection fraction, *HBR* high bleeding risk, *ARC-HBR* Academic Research Consortium for High Bleeding Risk, *J-HBR* Japanese version of HBR, *ACE-I* angiotensin-converting enzyme inhibitor, *ARB* angiotensin II receptor blocker, *CCB* calcium channel blocker

**Table 2:** Procedural and lesion characteristics

	Frail group (CFS ≥ 5)	Non-frail group (CFS < 5)	*p*-value
	(*N* = 38)	(*N* = 201)	
Indication of PCI			
Stenotic lesion ≥ 90%	22 (57.9%)	55 (27.4%)	0.001
Stenotic lesion > 50% plus functional ischemia assessment	16 (42.1%)	146 (72.6%)	
Fractional flow reserve or instantaneous wave-free ratio	7 (18.4%)	42 (20.9%)	
Scintigraphy	8 (21.1%)	76 (37.8%)	
Exercise stress testing	1 (2.6%)	28 (13.9%)	
PCI procedure (finalized devices)			
DES	36 (94.8%)	180 (89.5%)	0.28
DCB	1 (2.6%)	17 (8.5%)	
Others	1 (2.6%)	4 (2.0%)	
PCI access			
Radial	23 (60.5%)	156 (77.6%)	0.07
Femoral	10 (26.3%)	24 (11.9%)	
Brachial	5 (13.2%)	21 (10.5%)	
Contrast volume (mL)	80 (60–105)	85 (60–115)	0.25
Lesion location			
RCA	11 (29.0%)	62 (30.8%)	0.96
LAD	19 (50.0%)	99 (49.3%)	
LCX	7 (18.4%)	37 (18.4%)	
LMCA	1 (2.6%)	3 (1.5%)	
AHA/ACC lesion type			
A/B1	10 (26.3%)	77 (38.3%)	0.16
B2/C	28 (73.7%)	124 (61.7%)	
Number of the vessels			
Single	21 (55.3%)	118 (58.7%)	0.93
Double	10 (26.3%)	49 (24.4%)	
Triple	7 (18.4%)	34 (16.9%)	
LMCA or multivessel	18 (47.4%)	83 (41.3%)	0.49
Bifurcation lesion	7 (18.4%)	68 (33.8%)	0.06
Ostial lesion	3 (7.9%)	12 (6.0%)	0.65

Data are shown as median with interquartile range, and *n* (%)

*PCI* percutaneous coronary intervention, *DES* drug-eluting stent, *DCB* drug-coated balloon, *RCA* right coronary artery, *LAD* left anterior descending artery, *LCX* left circumflex artery, *LMCA* left main coronary artery, *AHA/ACC* American heart association/American college of cardiology

### Clinical outcomes

**Fig. 2:**
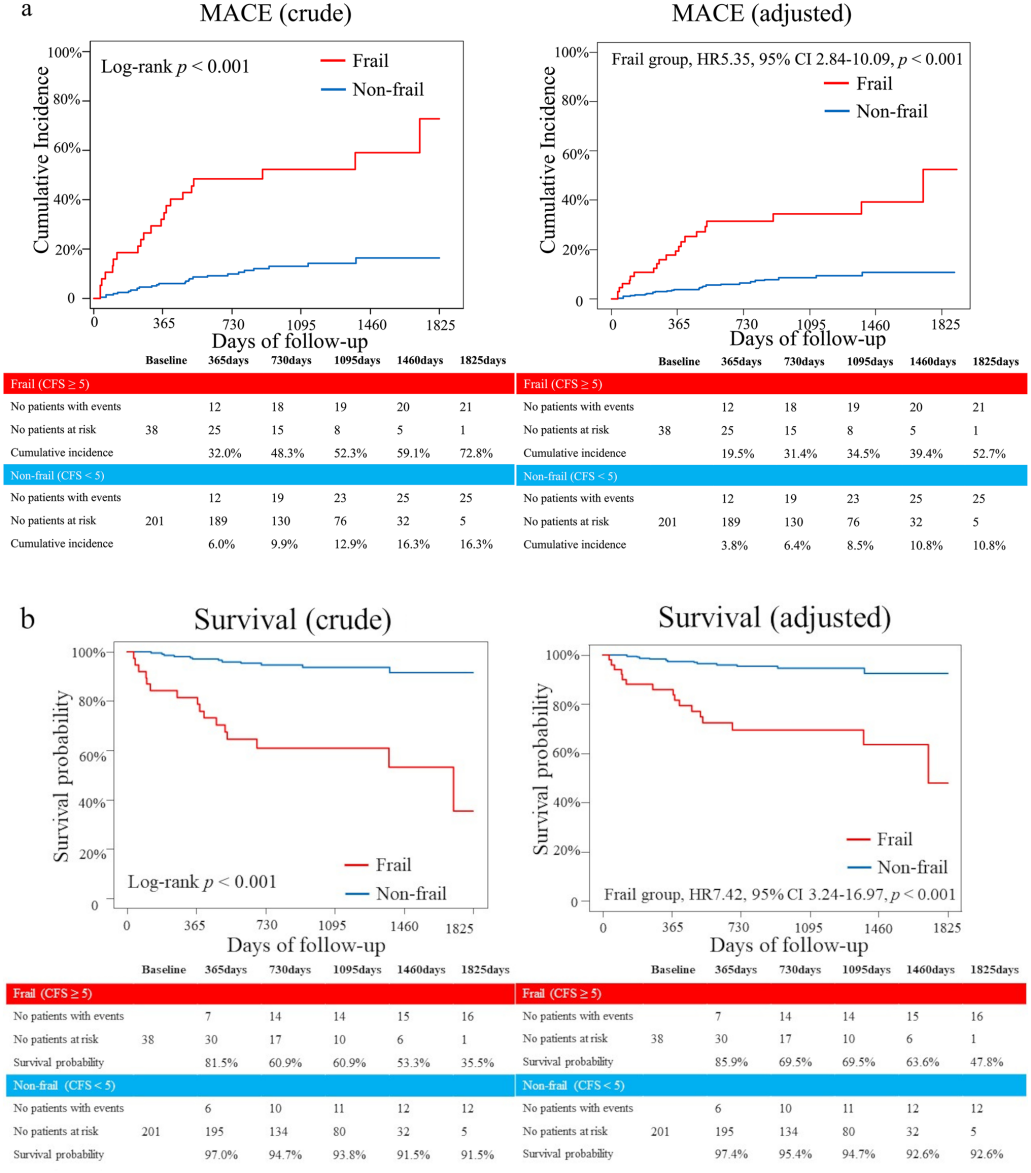
Kaplan–Meier analysis of the cumulative incidence of the primary outcome measure and survival probabilitybetween the two groups, the frail group (CFS ≥ 5), and the non-frail group (CFS < 5). **a** The primary outcome measure between the two groups. The crude Kaplan–Meier curve on the left side, and the Kaplan–Meier curve after adjustment for age and sex on the right side. The table below the adjusted Kaplan–Meier curve presents the number of patients at risk and patients with events in the unadjusted analysis. **b** Survival probability between the two groups. The crude Kaplan–Meier curve on the left side, and the Kaplan–Meier curve after adjustment for age and sex on the right side. The table below the adjusted Kaplan–Meier curve presents the number of patients at risk and patients with events in the unadjusted analysis. *MACE* major adverse cardiovascular event, *CFS* clinical frailty scale

**Fig. 3:**
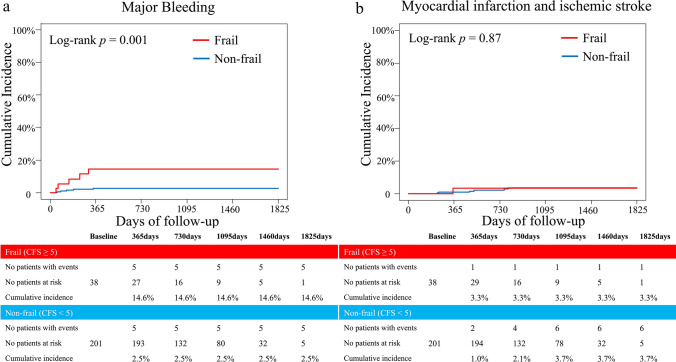
Kaplan–Meier analysis of the cumulative incidence of the secondary outcome measure between the two groups, the frail group (CFS ≥ 5), and the non-frail group (CFS < 5). **a** The secondary bleeding outcome measure between the two groups. **b** The secondary ischemic outcome measure between the two groups. *CFS* clinical frailty scale

The median follow-up period of the study population was 962 (607–1284) days. A total of 46 patients had MACE, 10 had major bleeding events and 7 had ischemic events during the follow-up period. Even after adjustment by age and sex, the primary outcome measure was significantly higher and the survival probability was significantly lower in the frail group than in the non-frail group (Fig. 2a and b). The table below the adjusted Kaplan–Meier curve presented the number of patients at risk and patients with events in the unadjusted analysis. In each subgroup analysis (65–74 years, ≥ 75 years), the cumulative incidence of MACE was significantly higher (Online Fig. 2 a, b, and Online Table 2a, b) and the survival probability was significantly lower in the frail group than in the non-frail group (Online Fig. 2c, d, and Online Table 2a, b). Each component of MACE is shown in Table 3. The cumulative incidence of all-cause death and heart failure requiring hospitalization was significantly higher in the frail group than in the other. In the evaluation of causes of all-cause death between the two groups, cardiac causes, and infectious disease were higher in the frail group, whereas malignancy was higher in the non-frail group (Online Fig. 3). Subgroup analysis stratified by age showed that patients aged 65–74 who were frail had significantly higher cardiovascular mortality than those without frailty. Additionally, frail patients aged 75 years or older had significantly higher non-cardiovascular mortality than non-frail patients (Online Table 2a and b). In contrast, the cumulative incidence of non-fatal MI and non-fatal stroke was comparable between the two groups. In the secondary outcome measure analysis, the cumulative incidence of persistent DAPT discontinuation was significantly higher in the frail group than in the non-frail group (Online Fig. 4). The Kaplan–Meier curves depicted that the cumulative incidence of major bleeding events was significantly higher in the frail group when compared with the non-frail group (Fig. 3a and Table 3), whereas the cumulative incidence of ischemic events (MI and ischemic stroke) was comparable between the two groups (Fig. 3b and Table 3).In the evaluation of causes of major bleeding events, the cumulative incidence of gastrointestinal bleeding and others (except gastrointestinal, intracranial, and access site bleeding) were significantly higher, whereas intracranial bleeding tended to be higher in the frail group than in those not frail (Table3). Subgroup analysis stratified by age (65–74 years and ≥ 75 years) showed that the cumulative incidence of major bleeding events was significantly higher in the frail group at an advanced age (≥ 75 years), but was similar between the two groups at age 65–74 years (Online Fig. 5a, b, and Online Table 2a, b). The cumulative incidence of ischemic events was similar between the two groups regardless of age (Online Fig. 5c, d, and Online Table 2a, b).

**Table 3:** Clinical outcomes between the frail and non-frail group

	All	Frail group	Non-frail group	*p*-value
	(*N* = 239)	(*N* = 38)	(*N* = 201)	
	No. patients with events (cumulative 2-year incidence; %)			
MACE	46 (15.9%)	21 (48.3%)	25 (9.9%)	< 0.001
All-cause death	28 (10.6%)	16 (39.1%)	12 (5.3%)	< 0.001
Cardiovascular death	9 (4.2%)	5 (15.8%)	4 (2.3%)	< 0.001
Non-cardiovascular death	19 (6.6%)	11 (27.7%)	8 (3.1%)	< 0.001
Non-fatal MI	4 (1.4%)	1 (3.3%)	3 (1.1%)	0.44
Non-fatal stroke	4 (1.4%)	1 (3.3%)	3 (1.1%)	0.45
Heart failure requiring hospitalization	12 (3.9%)	4 (9.3%)	8 (3.1%)	0.04
Major bleeding (BARC type 3 or 5)	10 (4.3%)	5 (14.6%)	5 (2.5%)	0.001
Gastrointestinal bleeding	4 (1.7%)	2 (5.8%)	2 (1.0%)	0.04
Access site bleeding	0 (0.0%)	0 (0.0%)	0 (0.0%)	1.00
Intracranial bleeding	2 (0.9%)	1 (3.6%)	1 (0.5%)	0.11
Others	4 (1.7%)	2 (6.0%)	2 (1.0%)	0.046
Ischemic event (MI and ischemic stroke)	7 (2.3%)	1 (3.3%)	6 (2.1%)	0.87

The number of patients with events counted during the entire follow-up. A cumulative 2-year incidence was estimated by the Kaplan–Meier method

*MACE* major adverse cardiovascular event, *MI* myocardial infarction, *BARC* Bleeding Academic Research Consortium

### Association between CFS and MACE

Table 4 shows the results of the univariate analysis of each baseline variable for primary outcomes. Univariate analysis revealed that CFS ≥ 5 was a significant factor associated with MACE (HR 5.86, 95% CI 3.27–10.51: *p*-value < 0.001). Furthermore, to evaluate whether high CFS (CFS ≥ 5) was the independent factor associated with MACE, multivariate analyses were performed as shown in Table 5. Pre-PCI frail (CFS ≥ 5) was the significant factor associated with the MACE after adjustment for variables including age, sex, DM, LVEF < 40%, LMCA or multivessel disease, and serum albumin < 3.5 g/dL (HR 4.27, 95%CI 1.86–9.80, *p*-value: < 0.001).

**Table 4:** Univariate analysis for the primary outcome

	Univariate	
	HR (95% CI)	*p*-value
Frail (CFS ≥ 5)	5.86 (3.27–10.51)	< 0.001
Advanced age (≥ 75 years)	1.95 (1.08–3.53)	0.03
Male sex	1.40 (0.69–2.81)	0.35
BMI < 18.5 kg/m^2^	1.87 (0.87–4.01)	0.11
History of PCI	0.82 (0.44–1.53)	0.53
History of OMI	1.03 (0.56–1.89)	0.93
History of stroke	1.41 (0.70–2.84)	0.34
History of heart failure	2.90 (1.56–5.39)	0.001
Hypertension	1.32 (0.59–2.95)	0.50
Dyslipidemia	0.63 (0.34–1.14)	0.13
Diabetes mellitus	1.22 (0.68–2.17)	0.51
Current-smoker	1.39 (0.70–2.73)	0.34
COPD	1.25 (0.53–2.95)	0.61
PAD	1.94 (1.04–3.64)	0.04
eGFR < 60 mL/min/1.73 m^2^	1.94 (1.06–3.57)	0.03
Hb < 11.0 g/dL	3.42 (1.87–6.24)	< 0.001
Serum albumin < 3.5 g/dL	6.69 (3.63–12.31)	< 0.001
LVEF < 40%	3.04 (1.51–6.13)	0.002
LMCA or multivessel	2.34 (1.29–4.23)	0.005

*HR* hazard ratio, *CI* confidence interval, *CFS* clinical frailty scale, *BMI* body mass index, *PCI* percutaneous coronary intervention, *OMI* old myocardial infarction, *COPD* chronic obstructive pulmonary disease, *PAD* peripheral artery disease, *eGFR* estimated glomerular filtration rate, *Hb* hemoglobin, *LVEF* left ventricular ejection fraction, *LMCA* left main coronary artery

**Table 5:** Multivariate analysis using Cox-proportional hazards regression models of frail (CFS ≥ 5) for primary outcome

	Multivariate analysis	
	Adjusted HR (95% CI)	*p*-value
Model 1		
Frail (CFS ≥ 5)	5.35 (2.84–10.09)	< 0.001
Age	1.02 (0.98–1.07)	0.26
Male sex	1.80 (0.87–3.73)	0.11
Model 2		
Frail (CFS ≥ 5)	3.93 (1.81–8.53)	< 0.001
Age	1.00 (0.96–1.05)	0.86
Male sex	1.87 (0.89–3.96)	0.10
DM	0.81 (0.43–1.52)	0.51
Serum albumin < 3.5 g/dL	4.12 (2.04–8.31)	< 0.001
Model 3		
Frail (CFS ≥ 5)	4.27 (1.86–9.80)	< 0.001
Age	1.00 (0.95–1.05)	0.99
Male sex	1.79 (0.85–3.79)	0.12
DM	0.69 (0.36–1.31)	0.26
LVEF < 40%	0.96 (0.40–2.35)	0.94
LMCA or multivessel	2.34 (1.18–4.62)	0.01
Serum albumin < 3.5 g/dL	3.63 (1.74–7.56)	< 0.001

*HR* hazard ratio, *CI* confidence interval, *CFS* clinical frailty scale, *DM* diabetes mellitus, *LVEF* left ventricular ejection fraction, *LMCA* left main coronary artery

Model 1 was adjusted for age and sex. Model 2 was adjusted for age, sex, DM and serum albumin < 3.5 g/dL. Model 3 was adjusted for age, sex, DM, LVEF < 40%, LMCA or multivessel disease, and serum albumin < 3.5 g/dL

## Discussion

The present study evaluated the association between pre-PCI CFS and clinical outcomes in patients with stable CAD who underwent elective successful PCI. The main findings in the present study were as follows: 1. Stable CAD patients who were frail (CFS ≥ 5) had worse clinical outcomes than those who were non-frail (CFS < 5) after PCI. 2; Stable CAD patients who were frail (CFS ≥ 5) experienced a higher incidence of major bleeding events than those not frail (CFS < 5) patients.

### Evaluation of frailty and MACE

In an aging society, the opportunities to treat older adults with cardiovascular disease and the number of frail patients are increasing. Therefore, assessing frailty is important when considering treatment strategies for older adults with cardiovascular diseases. A previous multicenter study based on CFS assessment reported that all-cause mortality at 2 years significantly increased as the severity of frailty increased in elderly ST-elevation myocardial infarction (STEMI) patients who underwent primary PCI [32]. Several studies indicated similar results in which the severity of pre-operative frailty was associated with poor clinical outcomes in patients with other cardiovascular diseases. These include critical limb ischemia patients who underwent bypass surgery [33]; heart failure patients who experienced implantation of cardiac implantable electric devices [34]; atrial fibrillation patients who undertook catheter ablation [35]; and CAD patients who underwent CABG [36]. However, there is limited data on the clinical outcomes of older frail patients with stable CAD who underwent elective PCI. In the present study, stable, frail CAD patients had worse clinical outcomes than the patients without frail despite the success of the procedure or other cardiovascular diseases. The reasons for this phenomenon might be as follows: 1. Frail patients are generally impaired in activities of daily living (ADL). A report indicates that patients with impaired ADLs are more likely to develop fatal diseases such as sepsis [37] and heart failure [38], which is attributed to an increased event rate in the frail group. Our study revealed that patients in the frail group had a higher incidence of heart failure requiring hospitalization and cardiovascular as well as non-cardiovascular death (including fatal sepsis cases) than those in the non-frail group. 2. Serum hemoglobin and albumin levels were significantly lower in the frail group than in the non-frail group. Poor nutritional status may also contribute to worse clinical outcomes. A previous report demonstrated that malnutrition was associated not only with the progression of atherosclerosis [39] but also with the weakness of the immune defense [40]. This explains the increased risk of cardiovascular and non-cardiovascular mortality in stable CAD patients of the frail group. These data suggest that pre-operative frailty assessment is crucial for predicting the clinical outcomes after the procedure. To improve the prognosis of patients with stable CAD complicated with frailty may not improve only with PCI but also with additional frailty improvement methods such as optimal medical therapy, exercise training, and nutritional intervention.

### Major bleeding event and CFS

Frailty is associated with increased bleeding events in patients with atrial fibrillation taking anticoagulation therapy [41] and those with STEMI who underwent primary PCI [42]. The present study is consistent with previous studies in that the cumulative incidence of major bleeding events at one year (14.6%) in the frail group was significantly higher than compared with that of the non-frail group (2.5%) [41, 42]. The possible explanations for this were as follows: 1. all frail patients have an HBR in the J-HBR criteria because of the inclusion of frailty in the J-HBR criteria. They also tend to have comorbidities such as anemia (hemoglobin < 11.0 g/dL), CKD, and anticoagulant use included in the J-HBR and ARC-HBR criteria [15, 16], and their J-HBR and ARC-HBR score tend to be higher. 2. frail patients are likely to have vulnerable perivascular support tissue due to decreased collagen fiber production [43]. The incidence of gastrointestinal bleeding events was significantly higher in the frail group despite the similarity of the proportion of proton pump inhibitor use. In contrast, access site bleeding was not observed in this study. The reason for this phenomenon is attributed to elective procedures in all patients and a relatively higher rate of trans-radial approach. Therefore, CFS should be evaluated before performing PCI in patients with stable CAD. Additionally, in patients with high CFS, a shorter duration of DAPT may reduce long-term bleeding events. When performing PCI in frail patients, the radial artery approach should be utilized to reduce the periprocedural bleeding risk. Furthermore, complicated PCI procedures should be avoided (e.g., avoiding full metal jackets for diffuse lesions and 2 stent strategy for bifurcation lesions) as much as possible to prevent a longer duration of DAPT.

### Study limitations

There are several limitations related to the study design and data collection methods. First, this was a retrospective non-randomized, small-sized, and single-centered study. Additionally, the association between CFS and clinical outcomes of stable CAD patients who underwent CABG or conservative therapy was not investigated. Because our hospital is a referral hospital type, the clinical outcomes of patients treated conservatively for stable CAD could not be compared with those of PCI patients because of insufficient follow-up and unknown clinical outcomes. Second, owing to discretional differences in surgeons, there is a possibility of selection bias regarding PCI procedures and medication, which may have affected the clinical outcome. However, as this study was conducted at a single institution, no significant differences concerning clinical treatment strategy were expected. Third, as CFS was investigated before the PCI procedure retrospectively from medical records, it may lack accuracy. Fourth, CFS was evaluated only once before PCI, and CFS changes were not assessed during the course of follow-up. However, evaluating CFS change over time may be more meaningful. Fifth, owing to the insufficiency of major bleeding and ischemic events, a multivariate analysis could not be performed to determine whether the frailty was associated with these conditions. Sixth, in the present study, functional ischemia assessment was more common in the non-frail group. Frail patients may have been less likely to undergo aggressive ischemic assessment for moderate stenosis, which may have affected the clinical outcome. In patients with stable CAD who underwent elective PCI, pre-PCI high CFS was independently associated with worse clinical outcomes. Hence, pre-PCI CFS assessment may be useful for risk stratification in patients with stable CAD.

## Supplementary Information

Below is the link to the electronic supplementary material.

Supplementary file1 (PDF 911 KB)

## Data Availability

The data that support the findings of this study cannot be shared publicly to protect the privacy of the study participants. The data will be shared by the corresponding author upon reasonable request.
