# Supplementary material for: Association of preoperative clinical frailty and clinical outcomes in elderly patients with stable coronary artery disease after percutaneous coronary intervention
Source: Heart Vessels. 2023 Jun 7;38(10):1205–17. doi: 10.1007/s00380-023-02276-3 (PMC10465392; doi:10.1007/s00380-023-02276-3)
Supplement: Supplementary file 1 — Supplementary file1 (PDF 911 KB) [file 380_2023_2276_MOESM1_ESM.pdf]

Association of preoperative clinical frailty and clinical outcomes in elderly patients with stable coronary artery disease after percutaneous coronary intervention

Heart and Vessels

Hirokazu Shimono, MD,[1] Akihiro Tokushige, MD.PhD,*[3,4] Daisuke Kanda, MD.PhD,[2] Ayaka Ohno, MD,[1] Masao Hayashi, MD,[1] Mana Fukuyado, MD,[1] Mitsumasa Akao, MD,[1] Mariko Kawasoe, MD,[1] Ryo Arikawa MD,[1] Hideaki Otsuji, MD,[1] Hideto Chaen, MD.PhD,[1] Hideki Okui, MD.PhD,[1] Naoya Oketani, MD.PhD,[1] and Mitsuru Ohishi, MD.PhD.FJCC[2,3]

[1]Department of Cardiovascular Medicine, Kagoshima City Hospital, Kagoshima, Japan

[2]Department of Cardiovascular Medicine and Hypertension, Graduate School of Medical and Dental Sciences, Kagoshima University, Kagoshima, Japan

[3]Department of Prevention and Analysis of Cardiovascular Diseases, Graduate School of Medical and Dental Sciences, Kagoshima University, Kagoshima, Japan

[4]Department of Clinical Pharmacology and Therapeutics, University of the Ryukyus School of Medicine, Okinawa, Japan

[*]**Corresponding Author:** Akihiro Tokushige

Department of Prevention and Analysis of Cardiovascular Diseases, Graduate School of Medical and Dental Sciences, Kagoshima University, 8-35-1 Sakuragaoka, Kagoshima City, Kagoshima 890-8520, Japan

Tel: +81-99-275-5318

Fax: +81-99-265-8447

E-mail: akihiro@med.u-ryukyu.ac.jp

Online Table 1. Univariate and multivariate logistic regression analysis for the frail (CFS ≥ 5)

	**Univariate**		**Multivariate**	
	**OR (95%CI)**	***p*-value**	**OR (95%CI)**	***p*-value**
Advanced age (≥ 75 years)	3.56 (1.67-7.58)	0.001	7.87 (2.60-23.86)	<0.001
Male sex	0.74 (0.35-1.57)	0.43		
BMI < 18.5 kg/m²	1.91 (0.70-5.18)	0.20		
History of PCI	0.80 (0.37-1.70)	0.56		
History of OMI	1.30 (0.64-2.66)	0.47		
History of stroke	3.21 (1.47-7.01)	0.003	5.63 (1.82-17.41)	0.003
History of heart failure	2.85 (1.29-6.30)	0.01	0.72 (0.23-2.26)	0.57
Hypertension	0.54 (0.24-1.18)	0.12		
Dyslipidemia	0.67 (0.32-1.41)	0.29		
Diabetes Mellitus	1.29 (0.64-2.58)	0.47		
Current-smoker	1.18 (0.50-2.79)	0.70		
COPD	1.94 (0.76-4.94)	0.17		
PAD	3.20 (1.49.-6.86)	0.003	2.18 (0.74-6.40)	0.15
eGFR< 60mL/min/1.73m²	1.80 (0.88-3.68)	0.11		
Hb < 11.0 g/dL	3.08 (1.42-6.71)	0.005	1.18 (0.38-3.62)	0.78
LVEF < 40%	5.16 (2.07-12.90)	< 0.001	5.49 (1.51-19.86)	0.01
LMCA or multivessel	1.28 (0.64- 2.57)	0.49		
Serum albumin < 3.5 g/dL	8.61 (3.69-20.10)	<0.001	11.06 (3.55-34.49)	<0.001

CFS; clinical frailty scale, OR; odds ratio, CI; confidence interval, BMI; body mass index, PCI; percutaneous coronary intervention, OMI; old myocardial infarction, COPD; chronic obstructive pulmonary disease, PAD; peripheral artery disease, eGFR; estimated glomerular filtration rate, Hb; hemoglobin, LVEF; left ventricular ejection fraction, LMCA; left main coronary artery.

Online Table 2a. Clinical outcomes between the frail and non-frail group aged 65–74 years old.

	All (N=130)	Frail group (N=11)	Non-frail group (N=119)	*p*-value
	No. patients with events (cumulative 2-year incidence; %)			
**MACE**	18 (12.2%)	5 (48.9%)	13 (9.0%)	<0.001
**All-cause death**	9 (6.0%)	4 (42.3%)	5 (3.0%)	<0.001
Cardiovascular death	5 (4.4%)	4 (42.3%)	1 (1.2%)	<0.001
Non-cardiovascular death	4 (1.6%)	0 (0.0%)	4 (1.8%)	0.63
**Non-fatal MI**	1 (0.9%)	0 (0.0%)	1 (1.0%)	0.81
**Non-fatal stroke**	2 (0.8%)	0 (0.0%)	2 (0.9%)	0.73
**Heart failure requiring hospitalization**	6 (4.7%)	1 (9.1%)	5 (4.3%)	0.41
**Major bleeding (BARC type 3 or 5)**	2 (1.6%)	0 (0.0%)	2 (1.7%)	0.68
Gastrointestinal bleeding	1 (0.8%)	0 (0.0%)	1 (0.9%)	0.78
Access site bleeding	0 (0.0%)	0 (0.0%)	0 (0.0%)	1.00
Intracranial bleeding	0 (0.0%)	0 (0.0%)	0 (0.0%)	1.00
Others	1 (0.8%)	0 (0.0%)	1 (0.8%)	0.76
**Ischemic event (MI and ischemic stroke)**	3 (1.7%)	0 (0.0%)	3 (1.8%)	0.68

The number of patients with events counted during the entire follow-up. A cumulative 2-year incidence was estimated by the Kaplan-Meier method. MACE; major adverse cardiovascular event, MI; myocardial infarction, BARC; Bleeding Academic Research Consortium.

Online Table 2b. Clinical outcomes between the frail and non-frail group aged ≥ 75 years old

	All (N=109)	Frail group (N=27)	Non-frail group (N=82)	*p*-value
	No. patients with events (cumulative 2-year incidence; %)			
**MACE**	28 (20.3%)	16 (48.2%)	12 (11.1%)	<0.001
**All-cause death**	19 (16.0%)	12 (38.0%)	7 (8.7%)	<0.001
Cardiovascular death	4 (4.0%)	1 (3.7%)	3 (4.0%)	0.88
Non-cardiovascular death	15 (12.4%)	11 (35.6%)	4 (5.0%)	<0.001
**Non-fatal MI**	3 (2.0%)	1 (4.8%)	2 (1.3%)	0.60
**Non-fatal stroke**	2 (2.1%)	1 (4.8%)	1 (1.4%)	0.30
**Heart failure requiring hospitalization**	6 (3.0%)	3 (9.3%)	3 (1.3%)	0.07
**Major bleeding (BARC type 3 or 5)**	8 (7.6%)	5 (20.5%)	3 (3.7%)	0.005
Gastrointestinal bleeding	3 (2.9%)	2 (8.4%)	1 (1.2%)	0.06
Access site bleeding	0 (0.0%)	0 (0.0%)	0 (0.0%)	1.00
Intracranial bleeding	2 (2.1%)	1 (5.3%)	1 (1.3%)	0.27
Others	3 (2.8%)	2 (8.4%)	1 (1.2%)	0.07
**Ischemic event (MI and ischemic stroke)**	4 (3.1%)	1 (4.8%)	3 (2.6%)	0.83

The number of patients with events counted during the entire follow-up. A cumulative 2-year incidence was estimated by the Kaplan-Meier method. MACE; major adverse cardiovascular event, MI; myocardial infarction, BARC; Bleeding Academic Research Consortium.

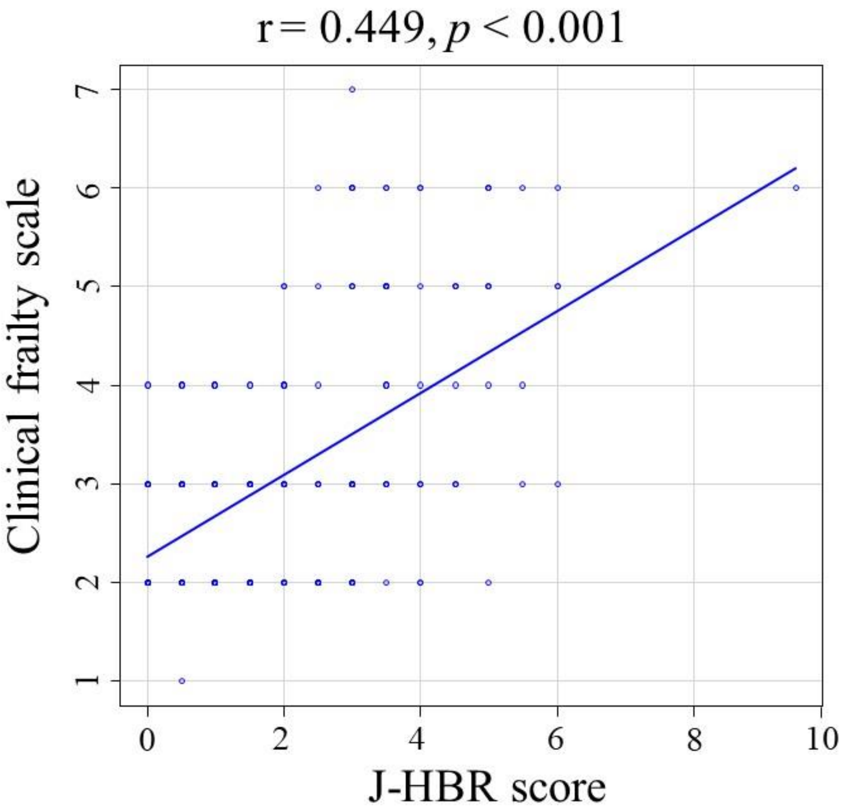

Online Figure 1. Correlation analysis between the Japanese version of high bleeding risk (J-HBR) score and the clinical frailty scale (CFS).

a

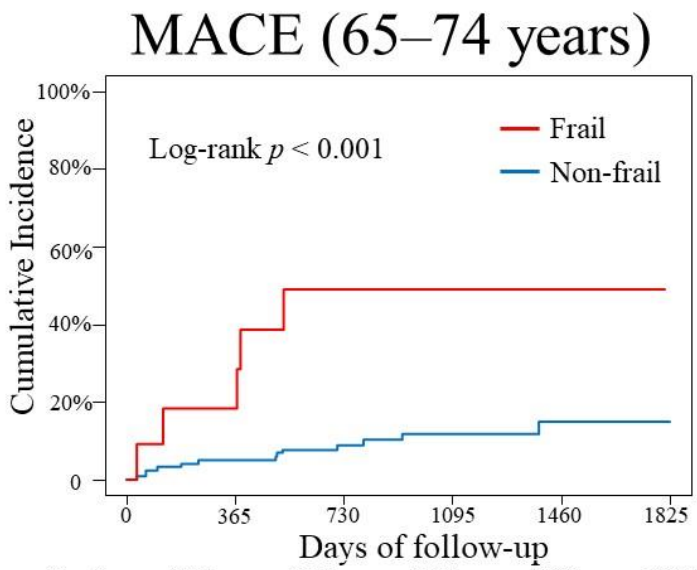

	Baseline	365days	730days	1095days	1460days	1825days
Frail (CFS ≥ 5)						
No patients with events		2	5	5	5	
No patients at risk	11	8	3	2	2	
Cumulative incidence		18.2%	48.9%	48.9%	48.9%	
Non-frail (CFS < 5)						
No patients with events		6	10	12	13	13
No patients at risk	119	113	76	45	21	4
Cumulative incidence		5.0%	9.0%	11.8%	14.9%	14.9%

b

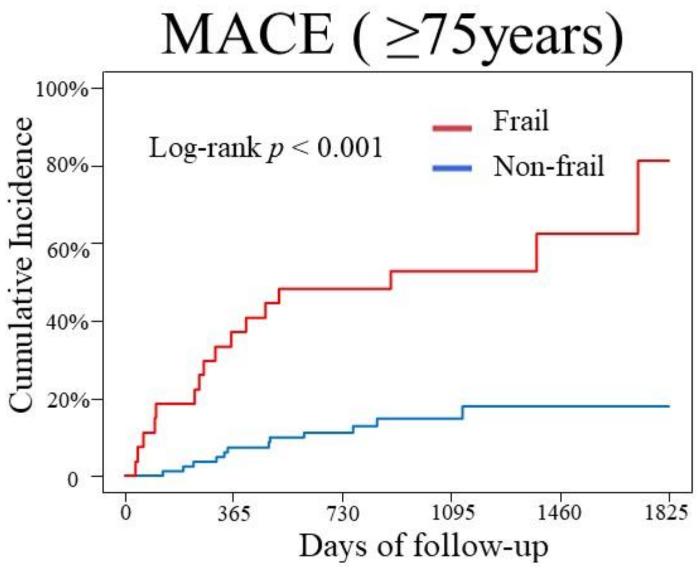

	Baseline	365days	730days	1095days	1460days	1825days
Frail (CFS ≥ 5)						
No patients with events		10	13	14	15	16
No patients at risk	27	17	12	6	3	1
Cumulative incidence		37.0%	48.2%	52.9%	62.3%	81.1%
Non-frail (CFS < 5)						
No patients with events		6	9	11	12	12
No patients at risk	82	76	54	31	11	1
Cumulative incidence		7.3%	11.1%	14.6%	17.7%	17.7%

c

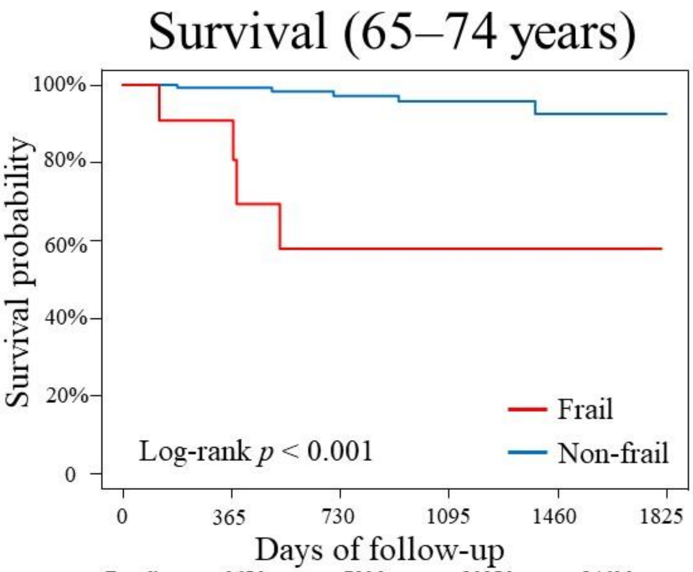

	Baseline	365days	730days	1095days	1460days	1825days
Frail (CFS ≥ 5)						
No patients with events		1	4	4	4	
No patients at risk	11	9	3	2	2	
Survival probability		90.9%	57.7%	57.7%	57.7%	
Non-frail (CFS < 5)						
No patients with events		1	3	4	5	5
No patients at risk	119	118	80	48	21	4
Survival probability		99.2%	97.0%	95.6%	92.2%	92.2%

d

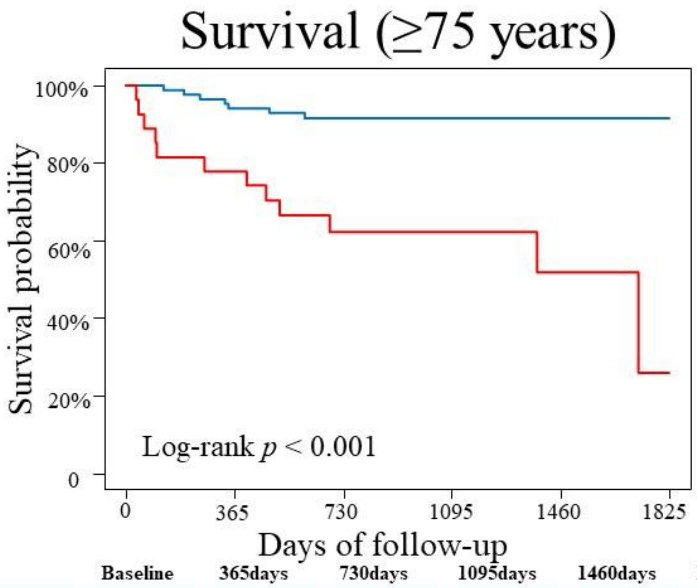

	Baseline	365days	730days	1095days	1460days	1825days
Frail (CFS ≥ 5)						
No patients with events		6	10	10	11	12
No patients at risk	27	21	14	8	4	1
Survival probability		77.8%	62.0%	62.0%	51.7%	25.9%
Non-frail (CFS < 5)						
No patients with events		5	7	7	7	7
No patients at risk	82	77	54	32	11	1
Survival probability		93.9%	91.3%	91.3%	91.3%	91.3%

Online Figure 2. Kaplan–Meier analysis of the cumulative incidence of the primary outcome measure and survival probability between the two groups, the frail group (CFS ≥ 5), and the non-frail (CFS < 5). (a) The primary outcome measure between the two groups (in patients 65–74 years old). (b) The primary outcome measure between the two groups (in patients ≥ 75 years old). (c) Survival probability between the two groups (in patients 65–74 years old). (d) Survival probability between the two groups (in patients ≥ 75 years old).

MACE; major adverse cardiovascular event, CFS; clinical frailty scale.

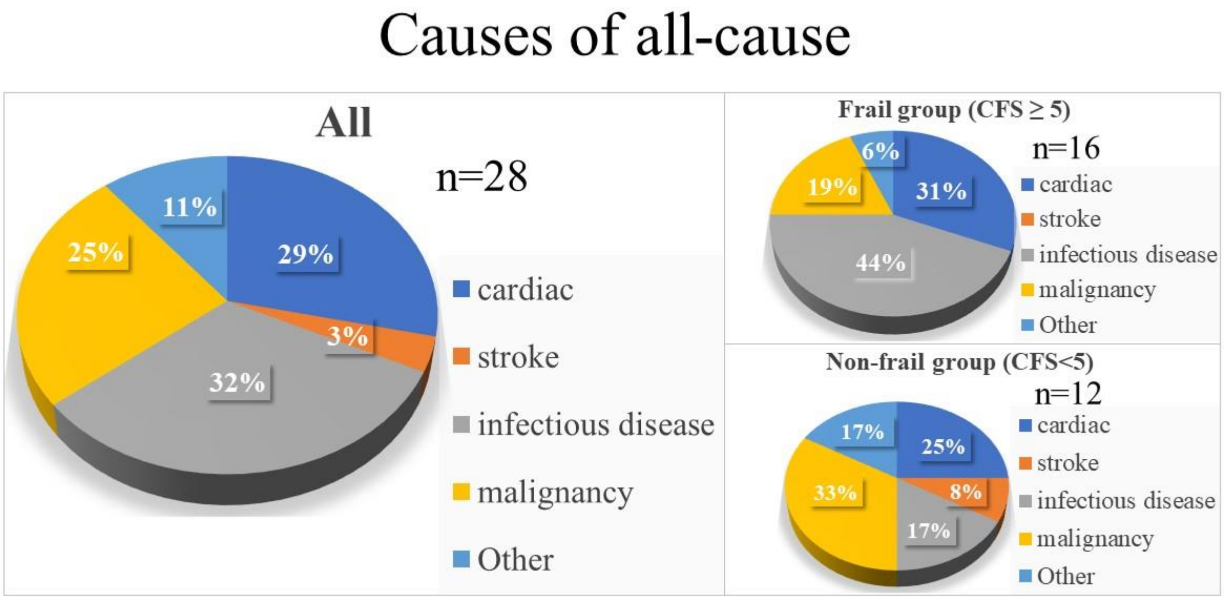

Online Figure 3. Cause of all-cause death between the two groups, the frail group (CFS ≥ 5) and the non-frail group (CFS < 5).

CFS; clinical frailty scale.

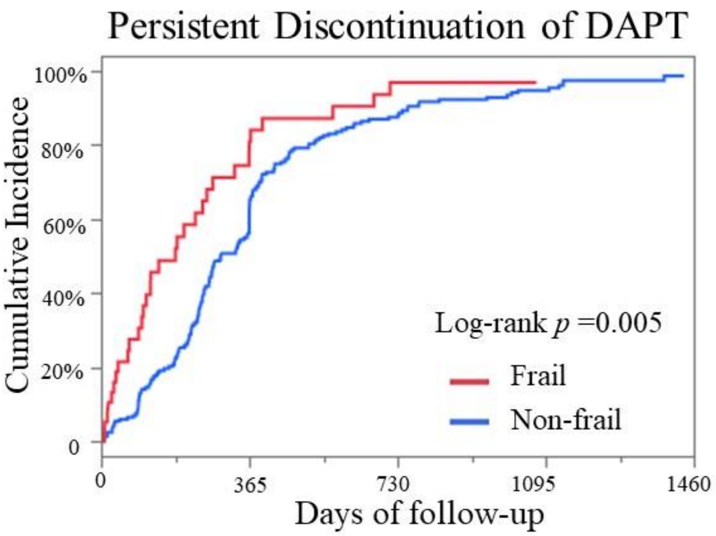

	Baseline	365days	730days	1095days	1460days
Frail (CFS ≥ 5)					
No patients with discontinuation		26	32		
No patients at risk	38	8	1		
Cumulative incidence		77.6%	96.8%		
Non-frail (CFS < 5)					
No patients with discontinuation		109	167	179	
No patients at risk	201	82	22	8	
Cumulative incidence		56.1%	87.6%	94.8%	

Online Figure 4. Kaplan–Meier analysis for the persistent discontinuation of DAPT between the two groups, the frail group (CFS ≥ 5) and the non-frail group (CFS < 5).

DAPT; Dual Antiplatelet therapy, CFS; clinical frailty scale.

a

Major Bleeding (65–74 years)

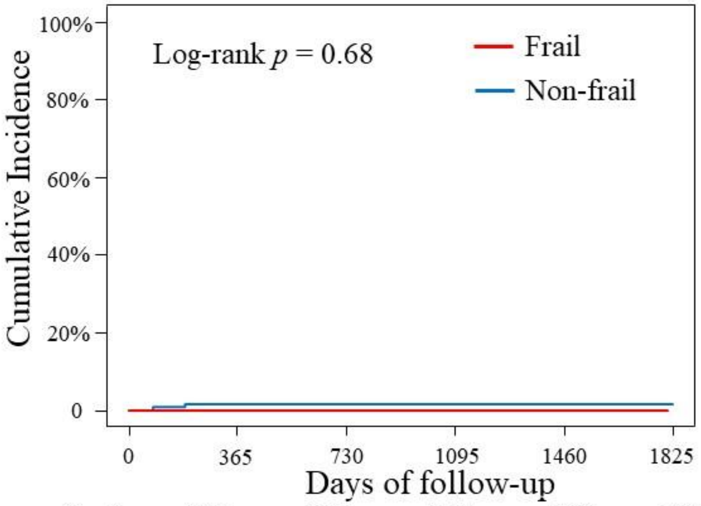

	Baseline	365days	730days	1095days	1460days	1825days
Frail (CFS ≥ 5)						
No patients with events		0	0	0	0	
No patients at risk	11	9	3	2	2	
Cumulative incidence		0.0%	0.0%	0.0%	0.0%	
Non-frail (CFS < 5)						
No patients with events		2	2	2	2	2
No patients at risk	119	116	78	48	21	4
Cumulative incidence		1.7%	1.7%	1.7%	1.7%	1.7%

b

Major Bleeding (≥75years)

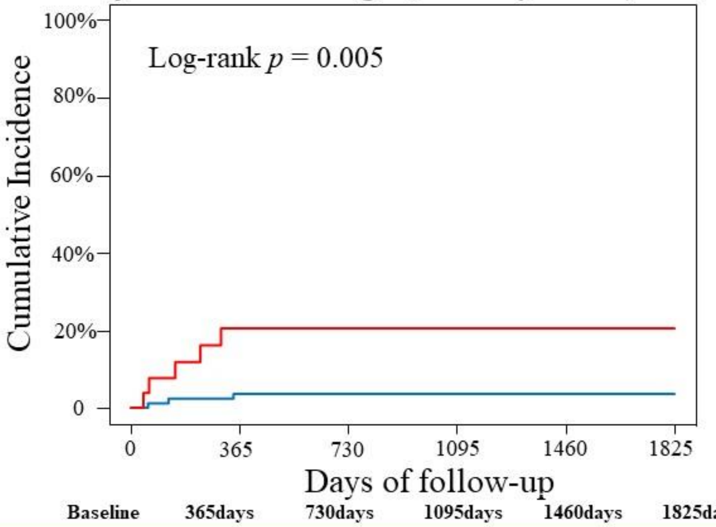

	Baseline	365days	730days	1095days	1460days	1825days
Frail (CFS ≥ 5)						
No patients with events		5	5	5	5	5
No patients at risk	27	18	13	7	3	1
Cumulative incidence		20.5%	20.5%	20.5%	20.5%	20.5%
Non-frail (CFS < 5)						
No patients with events		3	3	3	3	3
No patients at risk	82	77	54	32	11	1
Cumulative incidence		3.7%	3.7%	3.7%	3.7%	3.7%

c Myocardial infarction and ischemic stroke (65–74 years)

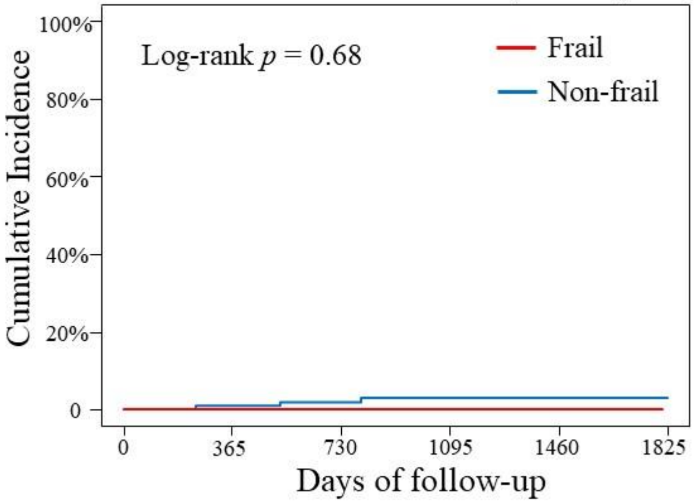

	Baseline	365days	730days	1095days	1460days	1825days
Frail (CFS ≥ 5)						
No patients with events		0	0	0	0	
No patients at risk	11	9	3	2	2	
Cumulative incidence		0.0%	0.0%	0.0%	0.0%	
Non-frail (CFS < 5)						
No patients with events		1	2	3	3	3
No patients at risk	119	117	78	47	21	4
Cumulative incidence		0.9%	1.8%	3.2%	3.2%	3.2%

d Myocardial infarction and ischemic stroke (≥75years)

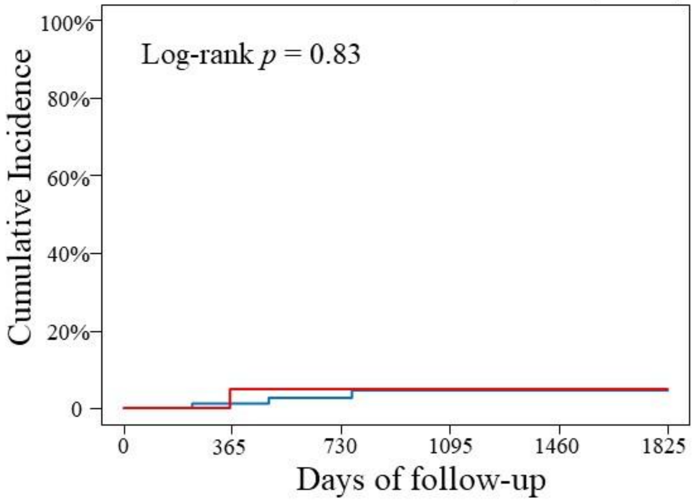

	Baseline	365days	730days	1095days	1460days	1825days
Frail (CFS ≥ 5)						
No patients with events		1	1	1	1	1
No patients at risk	27	20	13	7	3	1
Cumulative incidence		4.8%	4.8%	4.8%	4.8%	4.8%
Non-frail (CFS < 5)						
No patients with events		1	2	3	3	3
No patients at risk	82	77	54	31	11	1
Cumulative incidence		1.2%	2.6%	4.5%	4.5%	4.5%

Online Figure 5. Kaplan–Meier analysis of the cumulative incidence of the secondary outcome measure between the two groups, the frail group (CFS ≥ 5), and the non-frail (CFS < 5).

(a) The secondary bleeding outcome measure between the two groups (in patients 65–74 years old).

(b) The secondary bleeding outcome measure between the two groups (in patients ≥ 75 years old).

(c) The secondary ischemic outcome measure between the two groups (in patients 65–74 years old).

(d) The secondary ischemic outcome measure between the two groups (in patients ≥ 75 years old).

CFS; clinical frailty scale.
